# Transcriptome-Wide Identification, Evolutionary Analysis, and GA Stress Response of the *GRAS* Gene Family in *Panax ginseng* C. A. Meyer

**DOI:** 10.3390/plants9020190

**Published:** 2020-02-04

**Authors:** Nan Wang, Kangyu Wang, Shaokun Li, Yang Jiang, Li Li, Mingzhu Zhao, Yue Jiang, Lei Zhu, Yanfang Wang, Yingjie Su, Yi Wang, Meiping Zhang

**Affiliations:** 1 College of Life Science, Jilin Agricultural University, Changchun 130118, Jilin, China; wangnanwg@sina.com (N.W.); kangyu.wang@jlau.ed.cn (K.W.); lishaokun1168@163.com (S.L.); wky427@sina.com (Y.J.); lili910607@163.com (L.L.); zhaomingzhu0125@163.com (M.Z.); jiangyue285431@163.com (Y.J.); zhulei0916@163.com (L.Z.); suyj0923@163.com (Y.S.); 2 Research Center Ginseng Genetic Resources Development and Utilization, Changchun 130118, Jilin, China; yfwang2014@163.com; 3 College of Chinese Medicinal Materials, Jilin Agricultural University, Changchun 130118, Jilin, China

**Keywords:** *Panax ginseng*, GRAS transcription factor, DELLA sub-family, Evolutionary analysis, Gibberellin acid (GA), Stress response

## Abstract

GRAS transcription factors are a kind of plant-specific transcription factor that have been found in a variety of plants. According to previous studies, GRAS proteins are widely involved in the physiological processes of plant signal transduction, stress, growth and development. The Jilin ginseng (*Panax ginseng* C.A. Meyer) is a heterogeneous tetraploid perennial herb of the Araliaceae family, ginseng genus. Important information regarding the *GRAS* transcription factors has not been reported in ginseng. In this study, 59 Panax ginseng *GRAS* (*PgGRAS*) genes were obtained from the Jilin ginseng transcriptome data and divided into 13 sub-families according to the classification of *Arabidopsis thaliana*. Through systematic evolution, structural variation, function and gene expression analysis, we further reveal GRAS’s potential function in plant growth processes and its stress response. The expression of *PgGRAS* genes responding to gibberellin acids (GAs) suggests that these genes could be activated after application concentration of GA. The qPCR analysis result shows that four *PgGRAS* genes belonging to the DELLA sub-family potentially have important roles in the GA stress response of ginseng hairy roots. This study provides not only a preliminary exploration of the potential functions of the *GRAS* genes in ginseng, but also valuable data for further exploration of the candidate *PgGRAS* genes of GA signaling in Jilin ginseng, especially their roles in ginseng hairy root development and GA stress response.

## 1. Introduction

Ginseng is one of the most valuable medicinal plants in the Araliaceae family. Ginseng ingredients include saponins, polysaccharides, volatile ingredients, proteins, vitamins, and other compounds. The main active ingredient is ginsenosides. Asian regions, particularly China, Korea and Japan, are the major growing areas of ginseng. The Chinese ginseng is mainly distributed in Jilin Province and is commonly named as Jilin Ginseng. Ginseng has anti-thrombosis and anti-aging effects, prevents tumor angiogenesis and has other functions [1,2]. In recent years, the study of ginseng has become a hotspot for research.

Transcription factors (TFs) are important regulatory factors affecting growth and development, physiological processes and regulation of the network in higher plants. Additionally, TFs are necessary in the regulation and control of gene expression [3,4]. Under normal circumstances, transcription factors are combined with other proteins or DNA sequences to perform their special functions. In recent years, many transcription factor families have been discovered, such as the WRKY (WRKYGQK domain), MYB (V-myb avian myeloblastosis viral oncogene homolog), AP2/ERF (Ethylene responsive element binding factor), bZIP (Basic region/leucine zipper motif) and GRAS (GAI RGA and SCR) families [5,6,7,8]. GRAS TFs are named after the acronyms of three members that were initially identified, GAI (GIBBERELLIN-ACID INSENSITIVE), RGA (REPRESSOR of gai1-3) and SCR (SCARECROW) [9,10]. Typically, the GRAS protein is composed of 400–770 amino acid residues [11,12]. GRAS proteins possess a highly conserved sequence at the end of the carboxyl terminus which contains several ordered motifs LHRI, VHIID, LHRII, PFYRE and SAW [13,14]. Among these conserved sequences, the VHIID sequence, which is essential for the interaction between proteins, is located in the middle of the leucine-rich LHR I motif and LHR II motif [15]. PFYRE and SAW may be associated with the structural integrity of the GRAS family. There is a significant difference in the amino terminus motif in the GRAS, which may determine the functional specificity of the GRAS proteins. In accordance with the length and difference of amino terminus in Arabidopsis thaliana, the GRAS proteins were divided into 17 sub-families [14]. The 17 sub-families were named as *LlSCL* (lilium longiflorum scarecrow-like), *PAT* (phytochrome A signal transduction), *DELLA* (the DELLA motif containing protein), *HAM* (hairy meristem), *LS* (lateral suppressor), *SCL3* (scarecrow-like 3), *SCR* (scarecrow), *SHR* (short root), *DLT* (dwarf and low-tillering), *SCL32* (scarecrow-Like 32), *SCL4/7* (scarecrow-like 4 and 7), *NAP1* (nodulation signaling pathway 1), *NSP2* (nodulation signaling pathway 2), *RAM1* (reduced arbuscular mycorrhization 1), *RAD1* (required for arbuscule development 1), *SCLA* (scarecrow-like-A) and *OG-MIG1* (mycorrhiza-induced GRAS), respectively [9,16]. LISCL is involved in the regulation of microspore occurrence in anther by binding with the meiosis-associated promoter [2]. According to previous studies, the PAT of GRAS proteins has been involved in the early stages of photosensitive phytochrom A signaling transduction [17]. According to the previous study, we found that the HAM family was involved in the development of apical meristem and leaf axils of meristem tissue [18]. The LS sub-family is mainly involved in the regulation of the axillary meristem and formation of tillers [19]. In addition, it was found that SCL3 control of GA homeostasis during root development is crucial. The SCR and SHR are two different sub-families, but both generally participate in the growth of root radial tissue by forming SCR/SHR complexes [20]. NSP1 and NSP2 genes regulate the expression of the tumor factor and participate in biosynthesis of strigolactone [21]. Moreover, the SCL4/7 sub-family can increase plant tolerance to salt and drought stress [22]. The main functions of the *DLT* genes are to participate in the Brazilianno steroid signal in rice and to change the induced dwarfization and low tillage phenomena [23]. According to reports, the RAM1 sub-family is involved in transduction of the root signal [16]. The RAD1 and SCL32 genes are essential for inducing upon mycorrhization [3,24]. Meanwhile, the MIG1 proteins participates in regulating the change of cellular morphology during the formation of plexus branches [25]. However, for SCLA sub-families, their functions have not been discovered yet. The DELLA protein is a repressor which participates in the gibberellin signal response [26]. GA is a plant growth regulator which can promote the growth and development of plants and has important biological functions [27].

Hence, the study of the GA biosynthesis pathway and signal transduction is a hot research topic at present. Although these *GRAS* families have functional characteristics in the model plants, idiographic functions of a large number of GRAS proteins have not been found.

In this study, the *PgGRAS* gene family was identified from the Jilin ginseng transcriptome database and studied from aspects of evolution, function and expression to further understand the important role of the *PgGRAS* family. Furthermore, tissue-specific expression patterns in *Panax ginseng* was investigated by qPCR (Real-time quantitative PCR), and the expression patterns of gibberellin acid (GA) treatment were detected. Since our study demonstrates that *PgGRAS* gene family participates in plant secondary metabolism, fundamental information for the further study on plant responses to GA is provided, and the role of the GRAS gene family in the Gibberellin regulation pathway has been further verified.

## 2. Materials and Methods

### 2.1. Identification of GRAS Domain Genes in the Whole Transcriptome

The newest HMM model for the *GRAS* transcription factor gene family (PF03514.11) was downloaded from the Pfam database (http://pfam.sanger.ac.uk/) [28]. Screening was performed in the Jilin ginseng transcriptome using HMMER 3.0 [29]. The Jilin Ginseng database used in this experiment is composed of 248,993 transcripts extracted from 14 tissues of 4-year-old ginseng [30,31]. Then, we analyzed the amino acid sequences using NCBI CD-Search (http://www.ncbi.nlm.nih.gov/Structure/cdd/wrpsb.cgi). Only sequences with the GRAS domain were thought to be the *PgGRAS* genes for the next analysis. In order to further understand the properties of PgGRAS proteins, we used Protparam (http://web.expasy.org/protparam/) to predict and analyze the chemical and physical properties of PgGRAS proteins [32].

### 2.2. Conserved Motifs Analysis and Evolutionary Analysis of GRAS Genes

We used the NCBI ORF Finder (http://www.ncbi.nlm.nih.gov/orffinder/) to find the GRAS genes with complete conserved domains. We made use of MEME (http://meme.nbcr.net/meme/) for conserved motif analysis [33]. We downloaded the sequence of the OsGRAS domain protein from the *Oryza sativa* genome database (http://rice.plantbiology.msu.edu/analyses_search_locus.shtml), the sequence of SlGRAS domain proteins from the *Solanum lycopersicum* genome database (http://solgenomics.net/search/locus) and the *AtGRAS* gene sequence from the *Arabidopsis thaliana* genome database (http://planttfdb.cbi.pku.edu.cn/). We constructed gene trees using the Maximum-Likelihood (ML) method of MEGA 7.0 (http://mega.co.nz/) software with 100 bootstrap replications [34]. The final gene tree was edited using the Evolview version 3.0 online web (https://www.evolgenius.info/evolview/#login) [35].

### 2.3. Gene Expression Pattern, GO Function Classification and Enrichment Analysis

In order to understand the expression pattern of genes, the expression patterns of genes were further analyzed. The heatmaps were constructed using the TBtools version 0.6673 software [36]. Blast2go (https://www.blast2go.com/free-b2g-trial) software was used to annotate *PgGRAS* transcription functions [37]. Then, Gene Ontology (GO) was used for further analysis of the annotation results. The enrichment of the number of *PgGRAS* transcriptions classified into each subcategory was tested by the Chi-square test method. 

### 2.4. Network Analysis of PgGRAS Transcripts Genes

The R programming language and software (http://www.rproje ct.org/) was used to calculate the correlation coefficient of Spearman. A gene co-expression network was constructed using BioLayout Express ^3D^ version 3.2 software [38].

### 2.5. Response of PgGRAS Genes to Different Concentrations of GA

Ginseng hairy roots were cultured in 1/2 MS liquid medium for 33 days with no hormones added. Then, we obtained the secondary generation to cultivate as 1.0 g ginseng hairy root in 1/2 MS liquid medium each bottle. The GA was added after 23 days of culture; that is, ginseng hairy roots were cultivated by six gradients of GA concentration, which included 0.0 μM as control, 2.5, 5.0, 7.5, 10.0 and 15.0 μM (four repetitions per concentration gradient) [39]. At each treatment, each group of treatments was repeated three times biologically. The result is three duplicate averages, and all processing experiments were carried out in separate periods of time. The fresh weight and dry weight of the hairy roots were weighed after 30 days with the untreated hairy root as the control. All these samples were immediately frozen in liquid nitrogen and stored at −80℃ until RNA extraction [40].

For qPCR analyses, the total RNA extraction of ginseng hairy roots used the improved TRIzol method based on the published manufacturer’s instructions. The quality and concentration of each RNA sample was determined using gel electrophoresis and the ScanDrop 2000 spectrophotometer (AJ, Germany). First-strand cDNA synthesis was performed using 2.00 μg of total RNA in a 20 μL reaction volume, according to the manufacturer’s instructions for RNA purification with HiFiScript gDNA Removal cDNA Synthesis Kit (ComWin, Beijing, China). The GADPH (GenBank Accession No. KF699323.1) [41] of *Panax ginseng* was used as a reference gene. The qPCR of relative fluorescence quantification was measured using the Applied Biosystems 7500 Real-Time System (ABI, USA) and the Ultra SYBR Mixture Kit (Low ROX) (ComWin, Beijing, China). The designed primers (Table 1) were used to detect the response of the *PgGRAS* genes under GA treatment using SYBR qPCR analysis. The relative expression of the 4 *PgGRAS* genes were determined by the 2^−ΔΔCt^ method [42] and all the samples of biological replicates and technical replicates were repeated three times.

## 3. Results 

### 3.1. Transcriptome-Wide Identification GRAS Gene Family in Ginseng

Although the *GRAS* gene family has been investigated in many studies, it has not been specifically studied in *Panax ginseng*. In order to ensure the accuracy of the extracted sequence, two methods were used to find the sequence in this experiment. Using the GRAS, the conserved domain (PF03514) has been investigated as the inquiry sequence to search *PgGRAS* genes by the Hmmer method [29]. In another way, the latest Hmm (PF03514.11) of the GRAS domain was used as a blast inquiry sequence to search directly in the Jilin ginseng Transcriptome Database [30,31]. Exercising these two methods, we searched the Jilin Ginseng Transcriptome Library and finally got 131 transcripts derivatives under 79 gene IDs (Tables S1 and S2). Only 59 of these transcripts’ derivatives were intact, of which five *PgGRAS* genes pertained to the DELLA sub-family, namely *PgGRAS44-04*, *PgGRAS48-01*, *PgGRAS50-01*, *PgGRAS68-01* and *PgGRAS19-01* of the *PgGRAS* genes in ginseng [43,44].

The transcripts’ sequence lengths of the *PgGRAS* genes are between 201 (*PgGRAS15-01*) to 3343 bp (*PgGRAS65-02*). Through the analysis of the physical and chemical properties of PgGRAS proteins, we found that the molecular weights ranged from 431.00 to 87,107.53 Daltons (Da). There are also significant differences in the theoretical PI values of predictions. The maximum theoretical PI value is 11.22 (*PgGRAS75-01*) and the minimum theoretical PI value is 4.37 (*PgGRAS02-01*). The hydrophilic range of PgGRAS proteins are from −0.652 (*PgGRAS69-04*) to 0.684 (*PgGRAS66-06*) (Table 2). Only 12 PgGRAS proteins have hydrophilicity greater than 0, so it can be inferred that most PgGRAS proteins are hydrophilic. There were 101 PgGRAS proteins in which the number of positive amino acids was higher than that of negative amino acids, while in 25 PgGRAS proteins, the content of negative amino acids was higher than that of positive amino acids. Additionally, there were five PgGRAS proteins in which the number of positive amino acids was equal to the number of negative amino acids. It is speculated that the number of positive amino acids is greater than the number of negative amino acids in most of PgGRAS proteins. The data observation shows that most PgGRAS proteins are stable, while there are still 22 unstable PgGRAS proteins, for the reason that its instability index is less than 40. Based on these results, it could be concluded that there are great differences in physical and chemical properties among PgGRAS proteins (Table 2). 

### 3.2. Conserved Motif Analysis and Systematic Analysis of PgGRAS Gene Family

**Figure 1:**
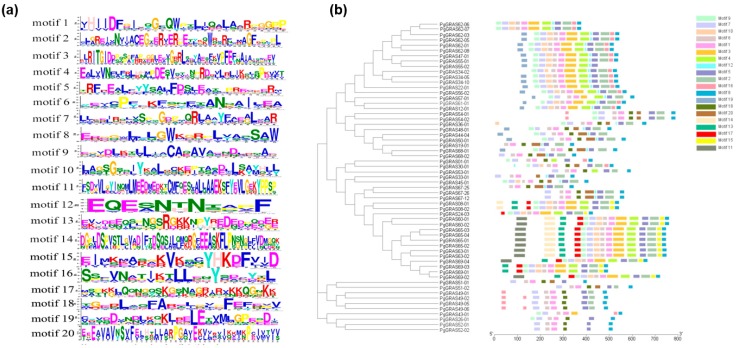
Analysis of conserved domains of PgGRAS proteins. (**a**) Conserved domains inferred from PgGRAS protein sequences are highlighted by MEME software. (**b**) Maximum-likelihood tree of PgGRAS proteins is shown on the left. Simultaneously display different subfamilies with different colors. Using different color motifs to reveal the conserved domain of the corresponding protein on the right.

By analyzing these results, we have obtained 131 transcripts’ sequences of *GRAS* genes in ginseng. For the sequence-structure study, we used the NCBI ORF Finder to search for the ORFs of 131 transcripts. We found that 59 transcripts had a complete ORF. However, the *GRAS* gene family has a unique conserved domain, in addition to other motifs. Additionally, there are five main motifs in conserved domains. The *GRAS* gene family has 20 motifs found throughout the literature (Figure 1a) [45]. Applying MEME for the analysis of these conserved motifs, we found that most of the transcripts have similar conserved motifs. The stability of the conserved domain is proven in Figure 1b. 

**Figure 2:**
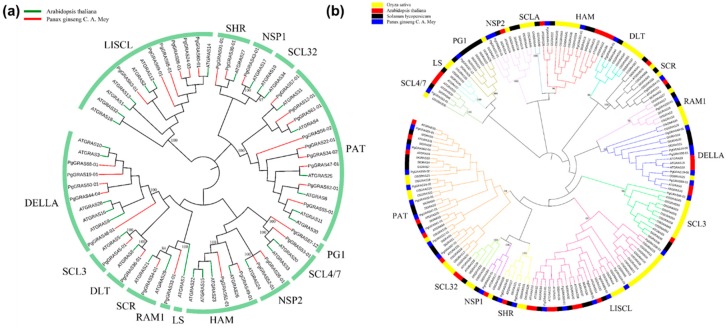
Evolutionary tree of the PgGRAS protein. (**a**) PgGRAS proteins were divided into 15 subfamilies according to the classification of *Arabidopsis thaliana* by using the maximum-likelihood (ML) method. Green, *Arabidopsis thaliana*. Red, *Panax ginseng* C. A. Meyer. (**b**) Evolutionary analysis of GRAS proteins in using the maximum-likelihood (ML) method to construct an evolutionary tree of *Solanum lycopersicum*, *Panax ginseng* C. A. Meyer, *Oryza sativa* and *Arabidopsis thaliana*. Members with the same color of the clade belong to the same subfamily. Members with the same color on the outer circle are of the same species. Black, *Solanum lycopersicum*. Red, *Arabidopsis thaliana*. Yellow, *Oryza sativa*. Blue, *Panax ginseng* C. A. Meyer.

In order to help with *PgGRAS* classification and decipher the evolution history of the *PgGRAS* gene family in ginseng, we built two evolutionary trees and applied 67 genes (Ginseng and Arabidopsis) (Figure 2a; Tables S2 and S3) and 172 genes (Ginseng, Arabidopsis, Tomato and Rice) (Figure 2b; Tables S2 and S3), respectively [46,47]. As shown in Figure 2, we identified a total of 15 sub-families of the *GRAS* genes. For ginseng, we identified nine *PgGRAS* genes in the PAT sub-families, six in the LISCL sub-family, five in the DELLA sub-families, two in the HAM sub-family, two in the SHR sub-family, two in the NSP2 sub-families, one in the SCL3 sub-family, one in the DLT sub-family, one in the NSP1 sub-families, one in the SCL4/7 sub-families, one in the RAM1 sub-family, one in the SCR sub-family and one in the PG1 sub-family (Figure 2a). The LLSCL sub-family has 13 members, and six *PgGRAS* genes (*PgGRAS08-1*, *PgGRAS24-03*, *PgGRAS60-01*, *PgGRAS63-01*, *PgGRAS65-01* and *PgGRAS69-01*) are highly homologous to *AtSCL9* (*AtGRAS27*) and may be associated with the regulation of the occurrence of microspores in meiosis [48,49]. The PAT sub-family has 15 members, nine of which (*PgGRAS12-01*, *PgGRAS22-01*, *PgGRAS34-02*, *PgGRAS47-01*, *PgGRAS55-01*, *PgGRAS56-02*, *PgGRAS57-01*, *PgGRAS61-01* and *PgGRAS62-01*) have a high degree of similarity to *AtPAT* (*AtGRAS30*) [50]. Based on previous studies, it was found that the Arabidopsis GRAS protein PAT was involved in the early stage of photosensitive pigment a signal transduction. These nine members may have similar functions. It has been reported that the HAM and LS families are involved in the development and regulation of axillary birth tissue, the apex meristem and leaf axils meristem of tiller formation, respectively [49]. The HAM sub-family consists of six members (*PgGRAS49-01*, *PgGRAS51-01*, *AtGRAS15*, *AtGRAS22*, *AtGRAS23* and *AtGRAS26*) and the LS sub-family consists of one member (*AtGRAS7*). The SCR and SHR sub-families are critical to the growth of radial tissue during the development of roots and buds. A total of three members (*PgGRAS01-01*, *PgGRAS30-01* and *PgGRAS54-01*) of the *GRAS* gene family belong to the SCR and SHR families and may be associated with the development of roots and buds [48]. 

The RAM1 sub-family contains only one ginseng gene (*PgGRAS33-01*), and the RAM1 protein is involved in inducing many AM (*Arbuscular mycorrhiza*)-related genes. Through the above, we can boldly infer that the *GRAS* gene is likely to be involved in the related process of AM. The SCL3 sub-family has two members (*AtGRA*S*5* and *PgGRAS45-01*) and one member in ginseng.

The *DLT* genes were involved in the brassinosteroid (BR) signaling, but only one gene (*PgGRAS36-01*) belongs to the DLT sub-family in ginseng [51]. NSP1 and NSP2 regulate the expression of genes associated with strigolactone biosynthesis, so that the root can be stimulated to mycorrhization by affecting hormones and their derivatives [3]. After analysis, it was found that three ginseng genes (*PgGRAS26-01*, *PgGRAS43-01* and *PgGRAS52-01*) belong to the NSP1 and NSP2 sub-families. Three genes (*AtGRAS20*, *AtGRA*S*33* and *PgGRAS53-01*) were observed in the evolutionary tree belonging to the SCL4/7 subfamily, which may be involved in the resistance system in the plant [2]. However, the SCL32 subfamily contains only two members (*AtGRAS19* and *AtGRAS34*) of the *Arabidopsis thaliana*, and there are no members of the SCL32 sub-family in the *Panax ginseng*. There was a unique subfamily found, namely PG1 (*PgGRAS67-12*), in ginseng (Figure 2a). As is known, the *GRAS* gene family has a subfamily DELLA that is involved in gibberellin signal responses and steady-state balance; the sub-family DELLA consists of 10 members (*PgGRAS68-01*, *PgGRAS19-01*, *PgGRAS50-01*, *PgGRAS44-04*, *PgGRAS48-01*, *AtGRAS10*, *AtGRAS3*, *AtGRAS28*, *AtGRAS16* and *AtGRAS9*), among which five members are from ginseng (Figure 2a). By understanding the DELLA sub-family, we can further study its influence on GA and speculate on its role in GA pathway [52].

According to the ML evolutionary tree, it can be seen that the genes of *Panax ginseng*, *Arabidopsis thaliana*, *Solanum lycopersicum* and *Oryza sativa* are mainly distributed in 13 sub-families (Figure 2b). No genes of ginseng were found in the SCLB sub-family. These results suggest that the origin of the *PgGRAS* gene family was before the separation of monocotyledons and dicotyledons [9,53].

### 3.3. Network Analysis of Gene Expression in PgGRAS Gene Family

**Figure 3:**
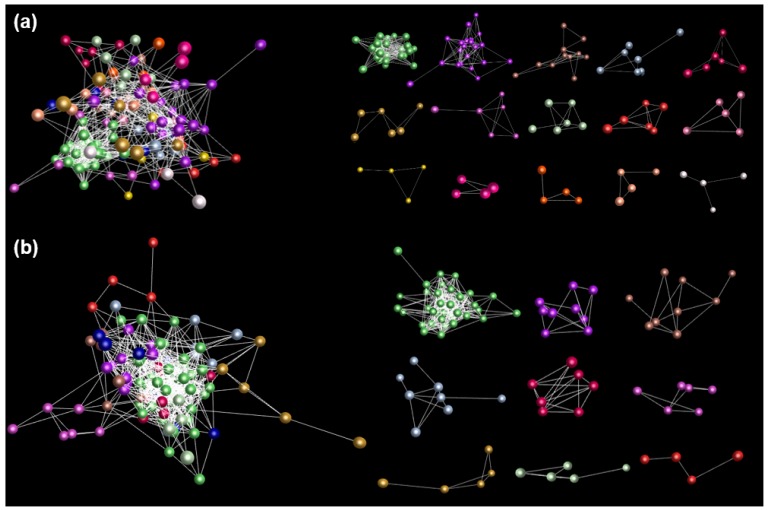
Network of the *PgGRAS* genes of the *PgGRAS* gene family. (**a**) Functional analysis of *PgGRAS* genes in 14 tissues from a 4-year-old ginseng plant. (**b**) Functional analysis of *PgGRAS* genes in 4-year-old ginseng roots of 42 ginseng farmers’ cultivars. Different color balls indicate the *PgGRAS* genes selected from different clusters of the *PgGRAS* gene family.

By means of the analysis of gene family genes, it can be said that there are great differences in expression and differentiation among functions of the family members of the gene. In order to observe the network of interactions among genes, we selected 131 transcripts expressed in 14 tissues from 42 farmers’ ginseng cultivars. Figure 3 shows a network of expression interactions among 131 transcripts (Figure 3a). These results show that with the functional differentiation of the *PgGRAS* gene family, the expression of its gene members is also different [54]. This phenomenon is not only observed within the gene family, but also sub-families. For the sake of checking whether the interaction among genes is associated with expression activities to some extent, we randomly sampled as the negative control the transcripts of 14 tissues of 4-year-old ginseng. Then, 131 *PgGRAS* transcripts tend to have associated expression and form a co-expression network (Figure 3b). These results show that the function and expression of the gene members of the *PgGRAS* gene family have been significantly differentiated. However, they do maintain a slight link between relevant expression and functional cooperation [55].

### 3.4. Expression Analysis and Functional Evolution of PgGRAS Gene Family

**Figure 4:**
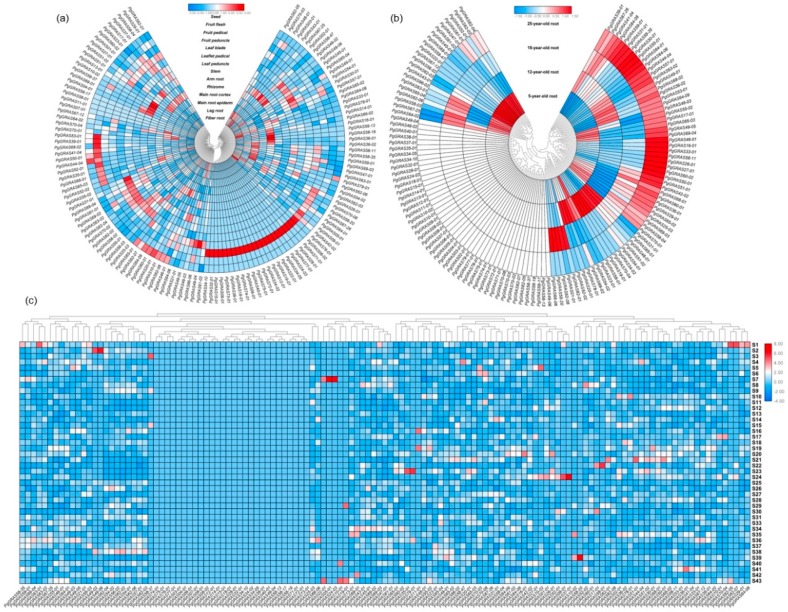
Relationship of the *PgGRAS* gene with their expression activities. (**a**) The expression spectrums of the 131 *PgGRAS* transcripts in 14 tissues of a 4-year-old ginseng plant. (**b**) The expression spectrums of the 131 *PgGRAS* transcripts in 5-, 12-, 18- and 25-year-old ginseng plants. (**c**) The expression spectrums of the 131 *PgGRAS* genes in 4-year-old roots of 42 ginseng farmers’ cultivars. The shades of color represent a relatively higher or lower level of expression.

**Figure 5:**
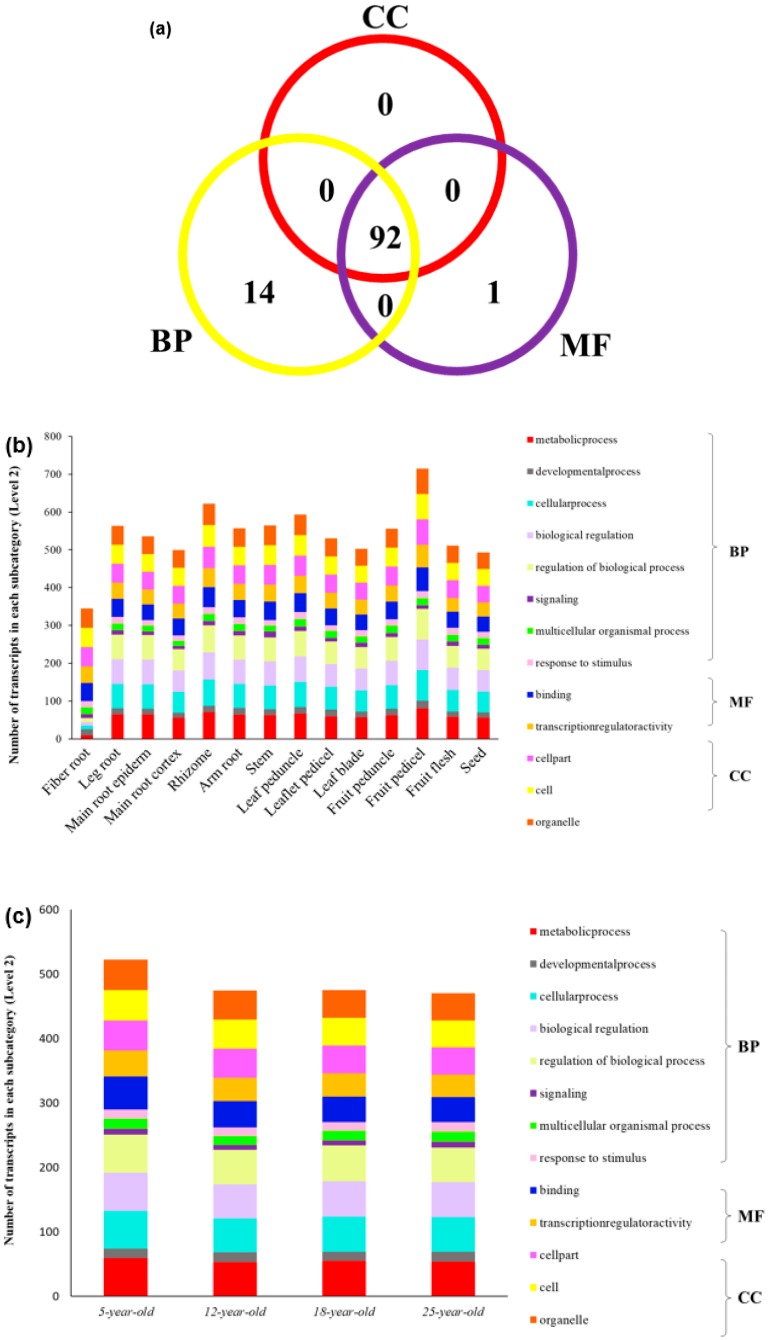

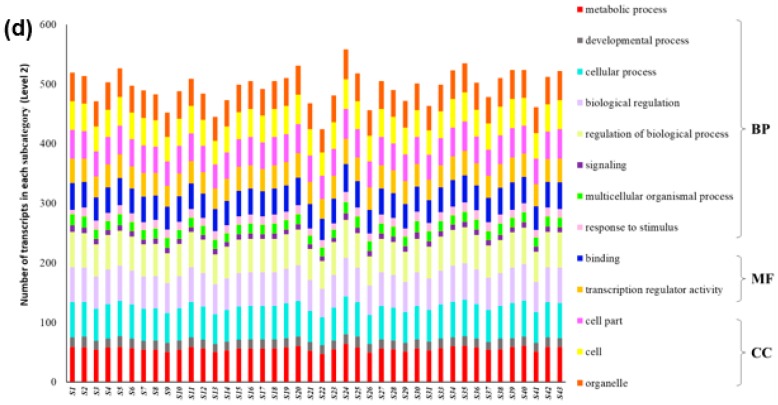
Functional categorization of the *PgGRAS* gene transcripts by Gene Ontology (GO). (**a**) Venn diagram of the functional categorization of the *PgGRAS* transcripts. Biological processes (BP), molecular function (MF) and cellular components (CC). (**b**) Variation of the functional categories of the *PgGRAS* transcripts among 14 tissues of a 4-year-old ginseng plant. (**c**) Variation of the functional categories of the *PgGRAS* transcripts among 5-, 12-, 18- and 25-year-old ginseng plants. (**d**) Variation of the functional categories of the *PgGRAS* transcripts among 4-year-old roots of 42 ginseng farmers’ cultivars.

**Figure 6:**
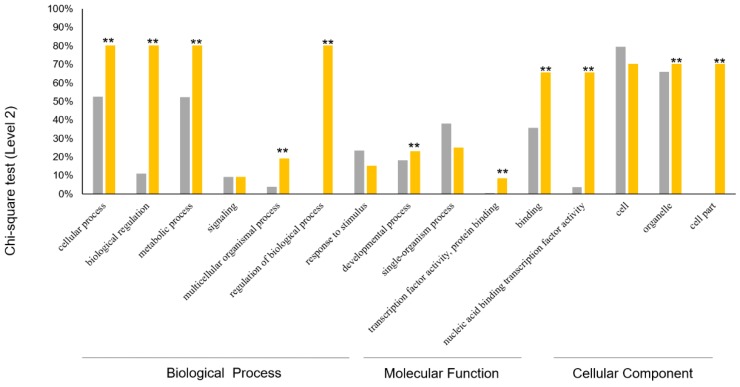
The Chi-square test was used to calculate the prediction and actual situation of the same function (Level 2) of the *PgGRAS* genes, further embodying the differentiation diversity of the *PgGRAS* genes function. Gray, the control group. Yellow, the *PgGRAS* genes group. **, very significant (*p* ≤ 0.01).

In order to further understand the expression of *PgGRAS* genes in ginseng, we used the selection of 131 ginseng transcripts for analysis: we analyzed the expressions of 131 transcripts and drew the heatmaps in the roots of 5-, 12-, 18- and 25-year-old ginseng plants. Fourteen tissues samples were taken from a 4-year-old ginseng plant; the 4-year-old roots of 42 genotypes were collected from 42 farmers and the expression profiles (Figure 4a–c and Table S4). At least 85 transcription profiles were found in 131 transcripts. At least 85 of 131 transcripts were expressed, and a number of *PgGRAS* genes had similar expression patterns. Heatmaps indicated that lots of *PgGRAS* genes not only had similar expression patterns, but also interactions. It is further shown that there is a network of interactions among genes to control gene expression. In order to improve the understanding of the functional relationships among gene sub-families, we used the Gene Ontology (GO) terms to analyze gene functions. According to the results, we found that 131 transcripts are divided into three main GO function categories—Biological Processes (BP), Molecular Function (MF) and Cellular Components (CC) [56]. Most of these transcripts have three functions at the same time, with only 15 genes having a single function (Figure 5a). These three terms have eight subcategories in Level 2—BP includes cellular process, biological regulation, metabolic process, signaling, multicellular organismal process, regulation of biological process, response to stimulus, developmental process and single-organism process; MF includes transcription factor activity, protein binding, binding and nucleic acid binding transcription factor activity; CC includes cell parts, cells and organelles. For more information about this study, the enrichment analysis was carried out. An enrichment analysis revealed that 11 subcategories in 15 subcategories were significantly enriched (*p* ≤ 0.01) in comparison with total transcriptome transcripts (Figure 6). Then, we performed a functional analysis of 131 *PgGRAS* genes that were found to have intact conserved domains (Figure 5b–d and Table S5). These results indicate that the gene function of each type of *PgGRAS* family had differentiated as they evolved.

### 3.5. Analysis of PgGRAS Genes Expression under Different Concentrations of GA

**Figure 7:**
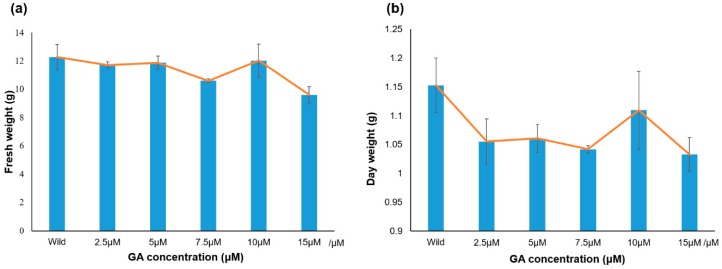
Fresh and dry weight of ginseng hairy roots were treated with different concentrations. (**a**) Fresh weight of ginseng hairy roots after treatment for 33 days. (**b**) Dry weight of ginseng hairy roots after treatment for 33 days. Error bars show the standard error between three replicates performed.

**Figure 8:**
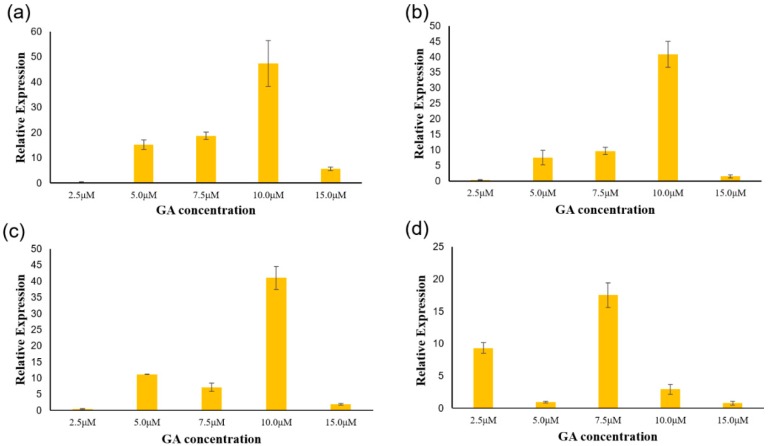
Relative expression levels of *PgGRAS* genes in different concentration GA treatments by qPCR. (**a**) Expression of *PgGRAS44-04* after GA treatment at different concentrations. (**b**) Expression of *PgGRAS48-01* after GA treatment at different concentrations. (**c**) Expression of *PgGRAS50-01* after GA treatment at different concentrations. (**d**) Expression of *PgGRAS68-01* after GA treatment at different concentrations. The expression level of the control sample was normalized to one. Error bars show the standard error between three replicates performed.

Plant hormones play an important role in the growth and development of plants, and GRAS transcription factors play an important role in the pathogenesis of metabolism and biosynthesis in plant growth [57,58]. Firstly, the growth and development of ginseng hairy roots treated with different concentrations of GA were measured. The fresh weight and dry weight of all the treatments reached their peak at the GA concentration of 10 μM, but were still less than those of control group (Figure 7). *GRAS* transcription factors are also involved in plant growth metabolic and biosynthetic pathways. After previous analysis, it was identified that *PgGRAS44-04*, *PgGRAS48-01*, *PgGRAS50-01*, *PgGRAS68-01* and *PgGARS19-01* belong to the DELLA sub-family, thus, it is speculated that it has the function of responding to gibberellin. After detection, the expression levels of these five genes were different under different concentrations of GA treatment. *PgGARS19-01* was not detected in the qPCR assay. Hence, no further analysis was performed. Here, we found that three genes—*PgGRAS44-04*, *PgGRAS48-01* and *PgGRAS50-01—*all reached their peaks of expression at 10.0 μM among treatments with different concentrations of GA (Figure 8a–c). The expressions of these three genes were gradually upregulated until GA reached the concentration of 10.0 μM, from which the expression levels decreased (Figure 8a–c). The expression level of *PgGRAS68-01* began to gradually decline after the concentration of 7.5 μM (Figure 8d). The results show that *PgGRAS44-04*, *PgGRAS48-01* and *PgGRAS50-01* had similar expression patterns. *PgGRAS44-04*, *PgGRAS48-01*, *PgGRAS50-01* and *PgGRAS68-01* all had different responses to exogenous GA addition. It is further demonstrated that the *PgGRAS* gene family can be applied to GA stress response.

## 4. Discussion

### 4.1. Function and Analysis of GRAS Transcription Factor Family

Since transcription factors play a very important role in plants, a large number of TFs are analyzed in many species [59]. However, the analyses of the *GRAS* gene family have been performed far less than those of other transcription factor families, and the study of the *GRAS* gene family in ginseng has not been reported until now. In this study, 131 transcripts of 79 genes from the Jilin ginseng transcriptome data of *Panax ginseng* were analyzed. The number of genes isolated from plants such as *Solanum lycopersicum* (53) and *Oryza sativa* (60) was similar, but higher than that of GRAS in *Arabidopsis thaliana* (34). The identification of these genes can further fill the gap of ginseng resources in GenBank. According to the gene tree, it can be observed that the *PgGRAS* gene family has been well clustered with the *GRAS* genes of *Arabidopsis thaliana*, *Oryza sativa* and *Solanum lycopersicum*. These results suggest that the origins of the *PgGRAS* gene family can be traced back to the division between monocotyledons (*Oryza sativa*) and dicotyledons (*Arabidopsis thaliana*, *Solanum lycopersicum* and *Panax ginseng*).

Through the functional analysis of *PgGRAS*, we found that the functions of the *PgGRAS* gene family have been largely differentiated. On the same level (Level 2), the *PgGRAS* gene family was categorized into at least eight GO functional subcategories and distributed across all three major GO functions, namely Biological Processes (BP), Molecular Function (MF) and Cellular Components (CC). There were great differences among the functions of the members of the *PgGRAS* gene family. However, according to the analysis, it was found that there were similar expression patterns among family members. Therefore, we can confirm that there is a network of co-expression among *PgGRAS* genes. Notwithstanding this, the function and expression activity of the *PgGRAS* genes have been greatly diversified. Most genes tend to have related expressions, and they also form a co-expression network, even if the correlation is limited. This indicates that members of the gene family maintain functional coordination or interaction to a certain extent. The formation of numerous mutual networks within the gene family provides a series of evidence for this inference, and it further confirmed the functional differentiation among members of the *PgGRAS* gene family.

### 4.2. Response of DELLA Sub-Family to Gibberellin and Its Effect on Ginsenosides

Expression analysis by qPCR revealed that four *PgGRAS* genes were involved in the GA regulatory pathway. According to previous reports, many families of transcription factors are involved in the regulation of hormones, and GA is involved in many aspects of plant growth and development, such as seed growth and development, stem elongation and flower development. After the comprehensive analysis of all the qPCR data (Figure 8), we found that four genes belonging to the DELLA sub-family (*PgGRAS44-04*, *PgGRAS48-01*, *PgGRAS50-01* and *PgGRAS68-01*) had significant changes in response to GA expression levels, suggesting that these genes could be involved in GA signaling and stress response.

GA as a plant hormone (gibberellin) is mainly synthesized in fruits or seeds, elongated stem ends and root organs of higher plants. Additionally, it promotes the germination of plant seeds, breaks dormancy and promotes stem and root growth. In addition, studies have found that GA and jasmonic acid (JA) interact with each other, which can play a synergistic role in plant development and an antagonistic role in plant defense. Predictions from other species indicate that GRAS proteins are an important component of the GA signaling pathway. In Arabidopsis, the DELLA protein acts as a repressor for GA response to plant growth [60,61]. Since studies have shown that the exogenous addition of JA can promote the content of ginsenosides in ginseng, it is speculated that there is a relationship between the synthesis of GA and ginsenoside content. The analysis of bioinformatics provides a basic information for studying the molecular regulation of GRAS proteins during ginseng development. This study provides ideas for future studies for the interaction between GA synthesis and the signal pathway and GA and ginsenoside synthesis in ginseng, and further improves ginseng resources.

## 5. Conclusions

In this study, 59 *GRAS* genes (*PgGRAS*) are found in Jilin ginseng and divided into 13 sub-families. These 59 *PgGRAS* genes have a correlation of expression and form a co-expression network to function. We identified that four *PgGRAS* genes belonging to the DELLA sub-family (*PgGRAS44-04*, *PgGRAS48-01*, *PgGRAS50-01* and *PgGRAS68-01*) had significant changes in expression levels in response to GA. The result of qPCR suggests that these genes can be involved in GA signaling and stress response. In summary, our results provide valuable data for further exploring the candidate *PgGRAS* genes of GA signaling in ginseng, especially their roles in ginseng hairy root development and stress response.

## Figures and Tables

**Table 1:** Sequences of *PgGRAS* and *GADPH* gene primers used in this study.

Gene Name	Forward Primer (5′-3′)	Reverse Primer (5′-3′)
*PgGRAS44-04*	GGCAATAGAAATGGGAATAAGCGAA	CTCTTGAGAATCGACAAGTACCAAC
*PgGRAS48-01*	TCGGACCTCAGTTACGTTGAC	TTCCTCCATCGCGGTTACAAC
*PgGRAS50-01*	CTGCACAAATATAGCACCACCG	ATTACCCCTTCCGACCAGAATG
*PgGRAS68-01*	GTGGAGGAACGGGTGATTGA	TCCGGCAGAGTCGTCTACTA
*GADPH*	GAGAAGGAATACACACCTGACC	CAGTAGTCATAAGCCCCTCAAC

**Table 2:** Physical and chemical properties of *PgGRAS* 131 genes in this study.

Name	Transcriptome ID	Nucleic Acid Length (bp)	Amino Acid Length	Molecular Weight (Da)	PI	Average of Hydropathicity
*PgGRAS01-01*	comp10366_c0_seq1	1634	431	431	5.45	−0.401
*PgGRAS02-01*	comp1049478_c0_seq1	338	34	3869.35	4.37	−0.2
*PgGRAS03-01*	comp1105291_c0_seq1	259	51	5983	11.01	−0.282
*PgGRAS04-01*	comp1121404_c0_seq1	312	85	9609.16	6.57	−0.085
*PgGRAS05-01*	comp1387617_c0_seq1	239	58	6767.62	4.87	−0.634
*PgGRAS06-01*	comp1596844_c0_seq1	258	78	8434.46	5.41	−0.145
*PgGRAS07-01*	comp17248_c0_seq1	210	68	7836.98	10.03	−0.35
*PgGRAS08-01*	comp17356_c0_seq1	2228	551	61,673.55	8.84	−0.377
*PgGRAS08-02*	comp17356_c0_seq2	2145	551	61,673.55	8.84	−0.377
*PgGRAS09-01*	comp1839771_c0_seq1	282	89	10,316.55	4.69	−0.321
*PgGRAS10-01*	comp19390_c0_seq1	258	54	6011.81	6.16	−0.369
*PgGRAS10-02*	comp19390_c0_seq2	221	39	4382.96	6.54	−0.292
*PgGRAS11-01*	comp2100322_c0_seq1	251	78	8584.94	4.6	0.306
*PgGRAS12-01*	comp212859_c0_seq1	2198	552	61,408.99	4.73	−0.291
*PgGRAS13-01*	comp22569_c0_seq1	1042	279	32,057.2	9.06	−0.142
*PgGRAS14-01*	comp2261495_c0_seq1	210	55	5954.67	5.22	−0.255
*PgGRAS15-01*	comp23356_c0_seq1	201	30	3357.14	10.31	0.023
*PgGRAS16-01*	comp25708_c0_seq1	865	266	29,992.15	5.74	−0.197
*PgGRAS17-01*	comp26247_c0_seq1	694	200	22,426.1	4.88	0.31
*PgGRAS18-01*	comp2660386_c0_seq1	202	39	4228.04	7.98	0.667
*PgGRAS19-01*	comp267650_c0_seq1	1725	353	38,544.56	5.69	−0.17
*PgGRAS20-01*	comp33784_c0_seq1	240	74	8432.86	8.8	−0.15
*PgGRAS21-01*	comp37210_c0_seq1	692	120	14,029.28	9.16	−0.512
*PgGRAS22-01*	comp38081_c0_seq1	1876	547	60,343.12	5.89	−0.362
*PgGRAS23-01*	comp40091_c0_seq1	956	229	24,803.16	4.85	0.083
*PgGRAS24-03*	comp418104_c0_seq1	1633	437	49,927.34	9.22	−0.45
*PgGRAS25-03*	comp44037_c0_seq3	782	72	8236.58	4.44	0.321
*PgGRAS26-01*	comp46234_c0_seq1	1723	527	60,102.48	4.61	−0.422
*PgGRAS27-01*	comp472334_c0_seq1	733	218	24,669.44	4.52	0.139
*PgGRAS28-01*	comp479040_c0_seq1	578	172	19,639.29	9.01	−0.61
*PgGRAS29-01*	comp50021_c0_seq1	652	129	14,932.18	6.19	−0.156
*PgGRAS30-01*	comp51501_c0_seq1	2097	523	59,299.2	5.99	−0.48
*PgGRAS31-01*	comp52182_c0_seq1	1105	244	26,552.45	4.9	−0.454
*PgGRAS32-01*	comp522134_c0_seq1	756	236	25,687.72	6.37	0.198
*PgGRAS33-01*	comp53859_c0_seq1	1411	358	59,615.92	5.74	−0.31
*PgGRAS34-10*	comp53978_c4_seq10	2272	541	59,954.61	5.71	−0.249
*PgGRAS34-02*	comp53978_c4_seq2	2346	536	40,253.29	6.61	−0.134
*PgGRAS34-05*	comp53978_c4_seq5	2194	541	59,615.92	5.74	−0.31
*PgGRAS35-01*	comp546436_c0_seq1	540	109	12,690.7	8.46	−0.376
*PgGRAS36-01*	comp54894_c0_seq1	2441	674	74,618.25	5.84	−0.396
*PgGRAS36-02*	comp54894_c0_seq2	915	198	22,784.94		−0.339
*PgGRAS37-01*	comp564672_c0_seq1	400	107	12,078.8	4.73	−0.128
*PgGRAS38-01*	comp572133_c0_seq1	458	141	15,201.03	5.07	−0.227
*PgGRAS39-01*	comp57286_c0_seq1	704	148	15,923.56	4.53	−0.376
*PgGRAS40-01*	comp578948_c0_seq1	309	91	10,079.48	11.16	−0.552
*PgGRAS41-04*	comp59054_c0_seq4	930	168	19,216.92	5.11	−0.066
*PgGRAS42-02*	comp59054_c1_seq2	345	76	8355.65	8.19	−0.095
*PgGRAS43-01*	comp59228_c0_seq1	2069	564	62,183.25	5.99	−0.521
*PgGRAS44-04*	comp59348_c0_seq4	1635	532	58,791.83	4.97	−0.15
*PgGRAS45-01*	comp59447_c0_seq1	1622	413	45,985.08	7.7	−0.094
*PgGRAS46-01*	comp61562_c0_seq1	2110	477	53,963.14	6.05	−0.161
*PgGRAS47-01*	comp61661_c3_seq1	2420	542	60,731.41	5.54	−0.335
*PgGRAS48-01*	comp62080_c0_seq1	2058	520	57,513.1	5.27	−0.128
*PgGRAS49-01*	comp62792_c0_seq1	2000	493	55,553.44	5.91	−0.126
*PgGRAS49-02*	comp62792_c0_seq2	2015	498	55,978.88	5.95	−0.114
*PgGRAS49-03*	comp62792_c0_seq3	1385	375	42,219.01	5.37	−0.177
*PgGRAS49-04*	comp62792_c0_seq4	1373	370	41,793.58	5.37	−0.194
*PgGRAS49-05*	comp62792_c0_seq5	1891	493	55,612.53	5.84	−0.129
*PgGRAS49-06*	comp62792_c0_seq6	1906	498	56,037.97	5.87	−0.117
*PgGRAS50-01*	comp62989_c0_seq1	2491	580	63,896.36	5.04	−0.243
*PgGRAS51-01*	comp63201_c1_seq1	1875	408	46,130.96	6	−0.103
*PgGRAS51-02*	comp63201_c1_seq2	2487	608	67,328.55	6.14	−0.225
*PgGRAS52-01*	comp63447_c0_seq1	1761	518	58,723.53	4.6	−0.275
*PgGRAS52-02*	comp63447_c0_seq2	1747	518	58,723.53	4.6	−0.275
*PgGRAS53-01*	comp63847_c0_seq1	2308	598	65,887.92	4.88	−0.277
*PgGRAS54-01*	comp64175_c2_seq1	3053	798	86,476.95	5.92	−0.284
*PgGRAS54-02*	comp64175_c2_seq2	3085	795	86,246.77	6	−0.289
*PgGRAS55-01*	comp64629_c0_seq1	2043	541	60,690.52	6	−0.343
*PgGRAS55-02*	comp64629_c0_seq2	1746	470	52,338.84	5.57	−0.339
*PgGRAS56-02*	comp64837_c0_seq2	3009	578	64,507.79	5.87	−0.421
*PgGRAS57-01*	comp65004_c0_seq1	2924	612	66,846.76	6.69	−0.294
*PgGRAS58-01*	comp65321_c0_seq1	916	143	16,880.14	6.51	−0.483
*PgGRAS58-11*	comp65321_c0_seq11	1094	143	16,880.14	6.51	−0.483
*PgGRAS58-13*	comp65321_c0_seq13	1958	143	16,880.14	6.51	−0.483
*PgGRAS58-17*	comp65321_c0_seq17	1254	143	16,880.14	6.51	−0.483
*PgGRAS58-18*	comp65321_c0_seq18	1760	143	16,880.14	6.51	−0.483
*PgGRAS58-20*	comp65321_c0_seq20	1087	143	16,880.14	6.51	−0.483
*PgGRAS58-03*	comp65321_c0_seq3	928	143	16,880.14	6.51	−0.483
*PgGRAS58-35*	comp65321_c0_seq35	1123	143	16,880.14	6.51	−0.483
*PgGRAS58-37*	comp65321_c0_seq37	1109	143	16,880.14	6.51	−0.483
*PgGRAS58-04*	comp65321_c0_seq4	1009	143	16,880.14	6.51	−0.483
*PgGRAS58-47*	comp65321_c0_seq47	449	123	14,595.54	5.93	−0.464
*PgGRAS59-01*	comp65321_c2_seq1	435	131	15,178.62	9.23	−0.41
*PgGRAS60-01*	comp65331_c0_seq1	2630	765	87,107.53	6.06	−0.462
*PgGRAS60-02*	comp65331_c0_seq2	2791	765	87,107.53	6.06	−0.462
*PgGRAS61-01*	comp65403_c0_seq1	2066	575	64,422.68	5.09	−0.347
*PgGRAS61-02*	comp65403_c0_seq2	1021	294	33,240.21	6.03	−0.14
*PgGRAS62-01*	comp65479_c0_seq1	2282	522	57,819.13	5.79	−0.053
*PgGRAS62-02*	comp65479_c0_seq2	2265	443	48,548.35	5.63	−0.03
*PgGRAS62-03*	comp65479_c0_seq3	3176	546	60,663.43	5.11	−0.229
*PgGRAS62-04*	comp65479_c0_seq4	2137	443	48,548.35	5.63	−0.03
*PgGRAS62-05*	comp65479_c0_seq5	3135	546	60,663.43	5.11	−0.229
*PgGRAS62-06*	comp65479_c0_seq6	3025	380	42,404.63	5.49	−0.03
*PgGRAS62-07*	comp65479_c0_seq7	2984	380	42,404.63	5.49	−0.03
*PgGRAS62-08*	comp65479_c0_seq8	2154	522	57,819.13	5.79	−0.053
*PgGRAS63-01*	comp66169_c0_seq1	3313	754	84,839.24	5.61	−0.446
*PgGRAS63-02*	comp66169_c0_seq2	2999	754	84,839.24	5.16	−0.446
*PgGRAS64-01*	comp66380_c0_seq1	2230	470	52,786.49	5.91	−0.185
*PgGRAS64-02*	comp66380_c0_seq2	2291	465	52,285.89	5.94	−0.221
*PgGRAS64-06*	comp66380_c0_seq6	2305	465	52,285.89	5.94	−0.221
*PgGRAS64-08*	comp66380_c0_seq8	1013	317	35,871.94	6.17	−0.38
*PgGRAS64-09*	comp66380_c0_seq9	2259	470	52,786.49	5.91	−0.185
*PgGRAS65-01*	comp67093_c0_seq1	2855	753	84,264.99	5.32	−0.437
*PgGRAS65-02*	comp67093_c0_seq2	3343	753	84,264.99	5.32	−0.437
*PgGRAS65-03*	comp67093_c0_seq3	2904	753	84,264.99	5.32	−0.437
*PgGRAS65-04*	comp67093_c0_seq4	3054	753	84,264.99	5.32	−0.437
*PgGRAS66-02*	comp67249_c0_seq2	498	75	8053.39	9.22	0.505
*PgGRAS66-06*	comp67249_c0_seq6	484	50	5415.45	9.4	0.684
*PgGRAS67-12*	comp67428_c0_seq12	1917	569	63,367.08	5.03	−0.123
*PgGRAS67-25*	comp67428_c0_seq25	1299	349	39,076.4	6.35	0.106
*PgGRAS67-26*	comp67428_c0_seq26	1891	569	63,367.08	5.04	−0.123
*PgGRAS68-01*	comp67501_c0_seq1	2041	540	59,906.01	5.03	−0.188
*PgGRAS68-02*	comp67501_c0_seq2	1733	361	39,883.54	5.83	−0.057
*PgGRAS69-01*	comp67516_c0_seq2	2051	499	58,454.58	8.26	−0.651
*PgGRAS69-02*	comp67516_c0_seq3	2633	726	83,814.41	5.91	−0.623
*PgGRAS69-03*	comp67516_c0_seq4	2099	499	58,454.58	8.26	−0.651
*PgGRAS69-04*	comp67516_c0_seq6	2538	671	77,840.89	6.02	−0.652
*PgGRAS70-01*	comp67518_c0_seq1	1172	123	14,161.05	6.28	−0.407
*PgGRAS70-02*	comp67518_c0_seq2	1081	129	15,078.18	7.63	−0.416
*PgGRAS70-03*	comp67518_c0_seq3	1415	123	14,161.05	6.28	−0.407
*PgGRAS70-04*	comp67518_c0_seq4	1055	129	15,078.18	7.63	−0.416
*PgGRAS70-08*	comp67518_c0_seq8	1298	129	15,078.18	7.63	−0.416
*PgGRAS71-01*	comp708716_c0_seq1	492	86	9590.63	5.49	−0.316
*PgGRAS72-01*	comp726689_c0_seq1	415	119	12,725.35	5.31	−0.224
*PgGRAS73-01*	comp753553_c0_seq1	363	78	7635.45	6.04	0.026
*PgGRAS74-01*	comp762664_c0_seq1	618	192	23,111.62	8.81	−0.505
*PgGRAS75-01*	comp774347_c0_seq1	439	60	7033.28	11.22	−0.522
*PgGRAS76-01*	comp866028_c0_seq1	430	68	7436.13	6.93	−0.503
*PgGRAS77-01*	comp876245_c0_seq1	1036	209	23,803.37	6.09	−0.293
*PgGRAS78-01*	comp913576_c0_seq1	443	58	6237.18	5.4	−0.084
*PgGRAS79-01*	comp933760_c0_seq1	420	132	14,868.61	5.93	−0.413

## Supplementary Materials

The following are available online at https://www.mdpi.com/2223-7747/9/2/190/s1, Table S1. The transcript sequences of the *PgGRAS* genes identified in this study. Table S2. The transcript protein sequences of the *PgGRAS* genes in this study. Table S3. The identified genes used as evolutionary tree for *PgGRAS* genes evolutionary analysis. Table S4. The expressions of *PgGRAS* transcripts (TPM) in 14 tissues, 42 farmers’ cultivars and four different year-old roots. Table S5. The classification, annotation and GO functional categorization of the *PgGRAS* gene transcripts.

## Additional Information

The sequences of the *PgGRAS* genes have been submitted at NCBI under BioProject RJNA302556.
